# Genetic Diversity and Antibiotic Resistance Paradigm of Enterobacterales in Animal-Derived Food Sources: A One Health Disquiet

**DOI:** 10.3390/pathogens14101040

**Published:** 2025-10-13

**Authors:** Ayesha Sarwar, Bilal Aslam, Muhammad Hidayat Rasool, Muhammad Shafique, Mohsin Khurshid, James Jacob Sasanya, Sulaiman F. Aljasir

**Affiliations:** 1 Institute of Microbiology, Government College University Faisalabad, Faisalabad 38000, Pakistan; ayesha.sarwar@gcuf.edu.pk (A.S.); drmhrasool@gcuf.edu.pk (M.H.R.); drmshafique@gcuf.edu.pk (M.S.); mohsinkhurshid@gcuf.edu.pk (M.K.); 2 Department of Veterinary Preventive Medicine, College of Veterinary Medicine, Qassim University, Buraydah 51452, Saudi Arabia; 3 International Atomic Energy Agency, 1400 Vienna, Austria; j.sasanya@iaea.org

**Keywords:** AMR, Enterobacterales animal-derived food, food producing animals, One Health

## Abstract

The indiscriminate use of antibiotics in food-producing animals serves as a major catalyst for the emergence of antibiotic-resistant infections. This study aimed to assess the genetic diversity and antibiotic resistance of Enterobacterales in animal-derived foods. A total of 905 animal-derived food samples, including meat, dairy, poultry, fish, and environmental sources, were collected from various locations in Pakistan. Isolates were confirmed through selective subculturing, morphological, biochemical, and MALDI-TOF analysis, followed by antibiotic susceptibility testing. Subsequently, PCR-based detection of antibiotic resistance genes and virulence-associated genes. Overall, a total of 263 (29.06%) Enterobacterales were identified, as follows: 58.55% (154/263) *E. coli*, 6.84% (18/263) *K. pneumoniae*, 21.29% (56/263) *P. mirabilis*, and 13.30% (35/263) *Salmonella* spp. Isolates showed a varying resistance pattern against different studied antibiotics, e.g., beta-lactams and inhibitors, ciprofloxacin, and tetracycline, while colistin and tigecycline remained most effective. All the isolates displayed an array of antibiotic resistance and virulence-associated genes. Particularly significant (<0.05) co-existence of *bla*_NDM_ and *mcr-1* was observed among the Enterobacterales isolated from various animal-derived foods. This study underscores the need to monitor Enterobacterales in animal-derived foods, especially in developing countries, to curb the spread of resistant pathogens and ensure effective food safety measures.

## 1. Introduction

Globally, antimicrobial resistance (AMR) poses a growing threat to human health and is recognized as a critical One Health concern. The interconnected framework of human, animal, and environmental health significantly contributes to the emergence, dissemination, and persistence of drug-resistant microbes locally and globally [1]. Drivers include the irrational use of antibiotics as growth promoters and prophylactics in animal husbandry, creating a self-perpetuating cycle of resistance. Consumption of contaminated animal-derived products disseminates resistant strains along with ARGs to humans, while drug residues and environmental reservoirs further amplify AMR across food-producing animals (FPAs), humans, and ecosystems [2]. Addressing this multisectoral challenge requires integrated surveillance spanning human health, veterinary, agricultural, and environmental domains.

Tackling AMR is essential for achieving Sustainable Development Goals (SDGs) through a One Health approach [3]. While FPAs are vital for food security (SDG 2), excessive antimicrobial use in livestock fuels AMR, jeopardizing human health (SDG 3), responsible consumption (SDG 12), and ecosystem integrity (SDG 15) [4]. Global antimicrobial use (AMU) in FPAs is projected to increase by 67% by 2030, driven by intensifying livestock production [5]. China, Brazil, India, the U.S., and Australia account for 58% of the global consumption, with Pakistan facing the largest relative increase (44%) [6]. Tetracyclines remain the most used antimicrobial class, though patterns vary regionally [7]. This trend exacerbates risks like allergic reactions, gut microbiome disruption, carcinogenesis, and the proliferation of antimicrobial-resistant bacteria (ARB) in food chains. Notably, global antimicrobial consumption in agriculture exceeds human medical use [8].

The National Action Plan (NAP) on AMR in Pakistan emphasizes reducing AMU and resistance. However, evidence-based policy implementation is hindered by a critical lack of reliable data on AMU trends and quantities in FPAs nationally [9]. Compounding this, rising demand for animal protein intensifies pressure on the food industry to increase yields while mitigating AMR transmission. Likely, resistant pathogens and ARGs spread through FPAs, contaminated food, environmental vectors (water, soil, and migratory species), and inadequate processing controls. Consequently, humans are exposed via direct contact or consumption of contaminated food products, leading to severe clinical outcomes [10].

Despite the recognized global threat of AMR within the One Health framework and alarming projections of increasing antimicrobial use in food-producing animals, particularly in countries like Pakistan facing significant relative increases, a critical knowledge gap persists. Specifically, there is a severe lack of comprehensive data from Pakistan quantifying the prevalence and diversity of antimicrobial resistance genes (ARGs), the detailed resistance profiles of Enterobacterales isolates circulating throughout the entire animal-derived food supply chain, i.e., from farm production to market. This absence of localized data significantly impedes the development and implementation of effective One Health interventions to mitigate AMR transmission.

Previously, we documented the distribution of shiga toxin-producing *E. coli* in the animal-derived food supply chain, which is a recognized public health threat from farm to fork [11]. However, in the current study, the specific objective was to bridge the gap by illustrating the genetic diversity and comprehensive antibiotic resistance profiling of Enterobacterales recovered from different sources and stages of the animal-derived food supply chain. Moreover, the detection of VAGs and ARGs was key to elucidating their potential as a significant animal-derived food-borne pathogen within this One Health-relevant ecosystem. Moreover, the correlation between the observed phenotypic resistance patterns and the underlying genotypic resistance markers was assessed. This study provides comprehensive data on the genetic diversity and resistance gene profiles of Enterobacterales across in animal-derived food supply chain in Pakistan, delivering crucial evidence to guide targeted One Health interventions against antimicrobial resistance transmission.

## 2. Materials and Methods

### 2.1. Ethical Approval and Study Settings

Ethical approval was taken from the Institutional Review Board (IRB) and the Ethical Review Committee (ERC) at the Government College University, Faisalabad (Ref No. GCUF/ERC/137, dated 3 February 2023). Written consent and prior permission were secured from all stakeholders before sample collection. All laboratory protocols were performed at the One Health AMR Laboratory (OH-AMR Lab) within the Institute of Microbiology, Government College University, Faisalabad. The Matrix-Assisted Laser Desorption/Ionization-Time of Flight (MALDI-TOF) analysis was conducted at the National Institutes of Health, M4QP + GW7, Islamabad, Pakistan.

### 2.2. Sampling, Categorization, and Transportation

Different animal-derived food samples were clustered and grouped into five categories: livestock products, poultry, environmental samples, fisheries, and dairy, which were further divided into subcategories as stated. Convenient random sampling was performed to collect total (n = 905) samples in sterile containers from various animal-derived food sources. These included livestock samples (n = 185), such as beef (80), mutton (80), and veal (25); poultry samples (n = 255), including chicken meat (90), cloacal/anal swabs (80), and droppings (85); environmental samples (n = 150) from slaughterhouses (25), open markets (25), manure sludge (25), dairy and poultry waste (50), and various transport vehicles (25); aquatic samples (n = 190) comprising fish (70), shrimps (45), market waste (25), transport vehicles (25), and raw fish waste (25); and dairy samples (n = 125), including raw milk (25), yogurt (25), dairy cream (25), cheese (25), and outlet waste (25).

Different sample sources included in the study were selected based on convenience, geographic coverage, market suitability, production systems, and public demand (e.g., seasonal variation, particularly in the case of fish that is consumed more in the winter season, buying capacity, cost, nutritional profile, etc.), whereas different sources like processed foods, imported food items, and clinically sick animals were excluded from the study. Market suitability and public demand regarding such expensive imported and processed food were significant factors for the exclusion of these sources. Moreover, due to the ongoing antibiotic treatment, diseased animals were excluded from the study.

The samples were preserved in buffer peptone water and transported in ice bags to the Institute of Microbiology’s laboratory for further investigation. The sample collection phase was extended from January 2022 to July 2023.

### 2.3. Isolation and Identification of Enterobacterales

The collected samples from various animal-derived food origins were first pre-enriched in tryptic soya broth (TSB) (Oxoid™, Basingstoke, UK) for all Enterobacterales and selenite broth (Oxoid™, UK) for *Salmonella* spp. at 37 °C for 24 h. Likewise, all the enriched Enterobacterales samples were streaked on nutrient agar (Oxoid™, UK), Klebsiella selective agar (HIMEDIA^®^, Maharashtra, India), and MacConkey (Oxoid™, UK) agar plates. Xylose lysine deoxycholate (XLD) agar (Oxoid™, UK), and *Salmonella Shigella* (SS) agar (Oxoid™, UK) for *Salmonella* spp. isolation. Incubation of Plates was performed aerobically at 37 °C for 24 to 48 h. Processing of the cultural and morphological characteristics of the samples was performed. Additionally, an API 20E kit (bioMérieux, Craponne, France) was used for the biochemical characterization as per manufacturer instructions.

### 2.4. MALDI-TOF

Identification of the isolates was confirmed using the MALDI-TOF-based VITEK^®^ MS V3.2 systems (bioMérieux, Craponne, France). All the procedures were carried out according to the manufacturer’s instructions. For matrix preparation, 0.01 µL of α-cyano-4-hydroxycinnamic acid (CHCA) was used. *Escherichia coli* ATCC™ 8739 served as the control strain. VITEK^®^ PICKMENIBS (bioMérieux, France) was employed to prepare the sample slides for both the VITEK^®^ MS analysis and the control. The ATCC™ strain and test isolates were inoculated onto the target plate in a circular motion. Afterward, 0.01 µL of CHCA was added to each spot, and the results were interpreted using the MYLA^®^ software version 4.9.1 (bioMérieux, Craponne, France).

### 2.5. Identification of VAGs Among Enterobacterales

Characterization of various VAGs in *E. coli* (*stx*1, *stx*2, *omp*T, *hyl*F, *pap*C, *eae*, *Amp*C, *tra*T, *fim*H, and *iss*), *K. pnumoniae* (*fim*H, *all*S, *ure*A, *Wab*G, *mrk*D, *iro*NB, *Kfu*, *rmp*A, *ent*B, and *uge*), *P. mirabilis* (*Uca*A, *Atf*A, *mrp*A, *hpm*A, *Zap*A, *Pt*A, *Ire*A, and *Fli*L), and *Salmonella* spp. (*Inv*A, *Spv*C, *Pef*A, *cdt*B, *Stn*, *Hil*A, and *iro*N) was performed using specific primers for PCR (Table S1). Gene JET Genomic DNA purification kit K0722 (Thermo Scientific™) was employed for DNA extraction following the mentioned procedure. Thermo-cycler:48 Biomerta™ (Göttingen, Germany) under specific conditions and respective annealing temperatures was used for PCR (Supplementary Materials).

### 2.6. Antimicrobial Susceptibility Testing

Antimicrobial susceptibility testing of Enterobacterales isolates (n = 235) was performed by the Kirby–Bauer disk diffusion method according to the 2021 CLSI instructions. *E. coli* ATCC™ 8739 was kept as a quality control during the experiment. CLSI instructions were followed to determine minimum inhibitory concentrations (MICs) using the broth microdilution method (BMD), except for colistin and tigecycline, which were monitored by EUCAST-CLSI and FDA instructions [12].

### 2.7. Rapid Polymyxin Test (RPT)

Colistin-resistant isolates (excluding *P. mirabilis*) were phenotypically confirmed using the Rapid Polymyxin Test (RPT), as previously described [12]. A stock solution of polymyxin was prepared in Mueller–Hinton Broth (MHB) using colistin (Oxoid™) to achieve a final concentration of 0.2 mg/mL. To prepare the Rapid Polymyxin NP solution (RPS), 6.25 g of MHB and 0.0125 g of phenol-red were mixed, and the pH was adjusted to 6.7. A sterile, filtered 1% D-glucose solution was also added to the RPS. In the initial step of the protocol, colistin was added to the RPS to yield a final concentration of 5 µg per 150 µL. The bacterial inoculum was prepared from freshly cultured *E. coli* colonies, which were suspended to match a 3.5 McFarland standard, following EUCAST guidelines. All the isolates (excluding *P. mirabilis*) that demonstrated growth in the presence of colistin were considered colistin-resistant.

### 2.8. Carbapenemase Nordmann-Poirel CLSI (CarbaNP CLSI) Test

The CarbNP test was conducted to confirm carbapenem-resistant isolates in accordance with CLSI guidelines [13]. A 100 µL volume of 20 mM Tris-HCl buffer was dispensed into two labeled Eppendorf tubes. Two solutions were then prepared: Solution A, consisting of 0.1 mM ZnSO_4_ and 0.5% phenol red indicator with the pH adjusted to 7.8, and Solution B, which was prepared by dissolving 6 mg/mL of imipenem in Solution A. Solution A was added to Tube 1, while Solution B was added to Tube 2. Both tubes were incubated at 37 °C for 2 h. A change to yellow color in Tube 2 indicated a positive CarbNP test, confirming the presence of carbapenemase activity.

### 2.9. Identification of ARGs Among Isolates

After performing resistance testing phenotypically, different ARGs in Enterobacterales were characterized, consisting of ESBLs (*bla*_CTX-M_, *bla*_SHV_, *bla*_TEM_, *bla*_OXA_, and *bla*_CMY_); MBLs (*bla*_NDM_, *bla*_KPC_, *bla*_OXA_, *bla*_VMP_, and *bla*_IMP_); and Qnrs (*qnr*S, *qnr*B, and *qnr*A), sulphonamide resistance genes (*sul*1 and *sul*2), tetracycline resistance gene (*tet*A), mobile colistin resistance (*mcr*-1 and *mcr*-2), and aminoglycoside acetyltransferases (acc). For this purpose, the genomic DNA purification kit, designated K0722 (Thermo Scientific, Waltham, MA, USA), was employed for DNA extraction, and PCR was carried out using specific primers (Supplementary Materials).

### 2.10. Statistical Analysis

Data elements were organized in Microsoft Excel (Office 365) spreadsheets for statistical analysis. Correlation and linear regression analyses were performed to evaluate interactions between variables from different sample sources and to quantify the strength of their relationships. To compare group means, an analysis of variance (ANOVA) was conducted to determine whether differences between source means were statistically significant, with a significance threshold set at *p* < 0.05.

## 3. Results

### 3.1. Isolation and Biochemical Identification of Enterobacterales

Overall, out of (n = 905), a total of 263 (29.06%) isolates were confirmed Enterobacterales. Among the confirmed Enterobacterales, a total of 58.55% (154/263) were identified as *E. coli*, whereas 6.84% (18/263) *K. pneumoniae*, 21.29% (56/263) *P. mirabilis*, and 13.30% (35/263) were detected as *Salmonella* spp., respectively.

**Figure 1:**
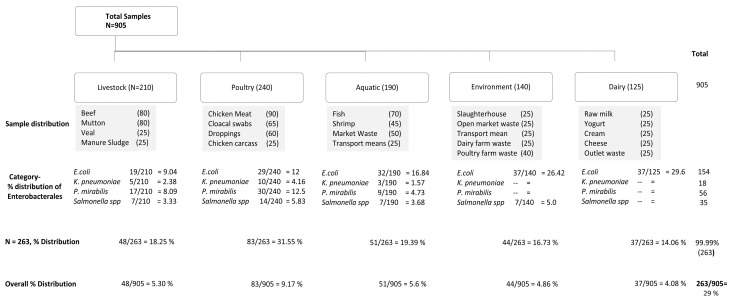
Overall distribution of Enterobacterales among various sample sources.

In case of 263 confirmed isolates, the distribution among different categories was recorded as follows: livestock 18.25%, poultry 31.55%, aquatic products 19.39%, environment 16.73%, and dairy 14.06% (Figure 1).

**Figure 2:**
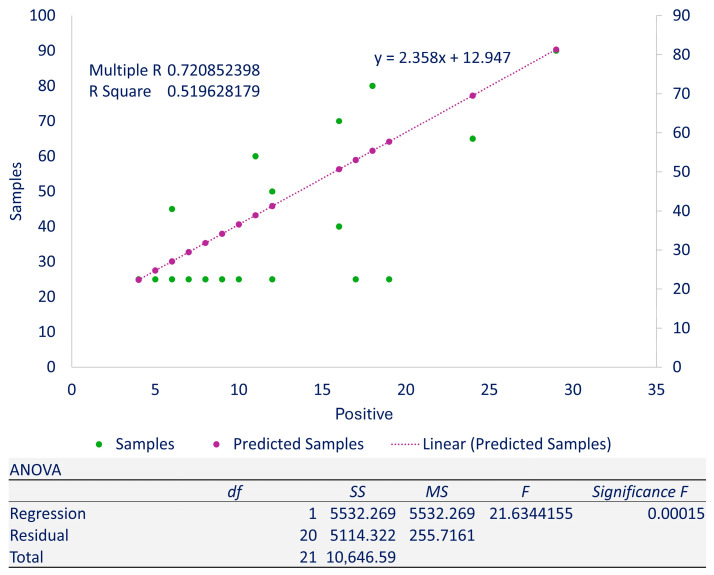
Linear regression plot showing the relationship between dependent variables and independent variables: the samples were taken as dependent variables while the isolates were taken as independent variables; additionally, ANOVA details are given.

Category breakdown along with statistical analysis is shown in (Table 1). Linear regression analysis showed a multiple R = 0.720852398 and R-squared 0.519628179 with a *p*-value = 0.00015 (Figure 2). Additional details along with isolate-wise distribution are also given (Supplementary Materials).

All the animal-derived food isolates were Gram-negative, capsule-forming rod colonies and utilized citrate as the sole carbon source. Growth of *E. coli* isolates on EMB agar appeared as a metallic green sheath, *K. pneumoniae* isolates on Klebsiella agar showed yellow, mucoid colonies, while *P. mirabilis* isolates grew on XLD agar as a pale-yellow colony with a black center, and lastly, *Salmonella* spp. isolates on Salmonella Shigella agar formed black central colonies. Moreover, they fermented various sugars, including glucose, lactose, arabinose, inositol, mannitol, mannose, sorbitol, and sucrose. The isolates tested negative for the Voges–Proskauer (VP) test except *K. pneumoniae*. In contrast, indole, H_2_S gas production, catalase, oxidase, and methyl red (MR) tests for all the Enterobacterales were performed. The API^®^ 20E strip (bioMérieux, France) was used for the identification of *E. coli*, *K. pneumoniae*, *P. mirabilis*, and *Salmonella* spp.

### 3.2. Antimicrobial Susceptibility Pattern of Enterobacterales from Animal-Based Foods

**Figure 3:**
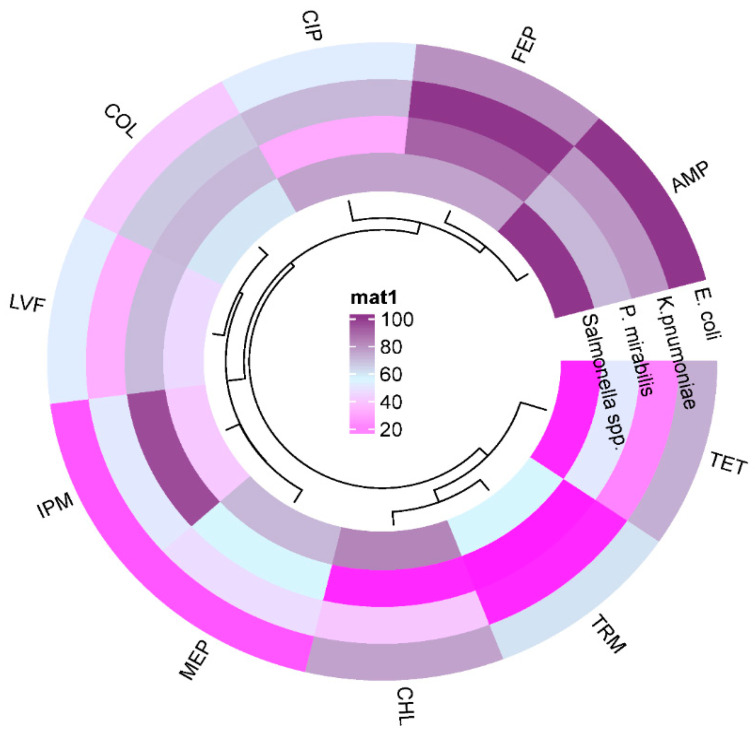
The circular cluster heat map showing the total resistance profile of various standard antibiotics against Enterobacterales isolates. The resistance pattern among Enterobacterales is depicted by color and cluster, illustrating the overall resistance percentages against antibiotics (AMP, FEP, CIP, COL, LVF, IPM, MEP, CHL, TRM, and TET). Resistance = dark purple, purple = sensitive, grayish green = intermediate.

Antimicrobial resistance profiling was determined by using the Kirby–Bauer disk diffusion method, and the Micro broth dilution assay was conducted for interpretation of results under the CLSI guidelines, 2020. The Enterobacterales selected for this study were *E. coli*, *K. pneumoniae*, *P. mirabilis,* and *Salmonella* spp. Antibiotics were used according to CLSI 2020 guidelines. Overall, among the tested Enterobacterales, highest resistance was seen in ampicillin (100%) and cefepime (90%), followed by chloramphenicol (82%), ciprofloxacin (75%), tetracycline (72%; except *P. mirabilis*), trimethoprim 60% and 60% colistin (except *P. mirabilis*), and 59% tigecycline, and the least was 4% in gentamicin (Figure 3).

### 3.3. ARGs Among Enterobacterales Isolates

Regarding the distribution of ARGs among 154 *E. coli* isolates, different ESBLs were determined as follows: *bla_CTX-M_* (52.59%), *bla*_SHV_ (16.88%), *bla*_TEM_ (35.71%), *bla*_OXA_ (7.14%), and *bla*_CMY_ (7.79%). In MBLs, *bla*_NDM_ showed (16.23%), followed by *bla*_OXA_ (8.44%), *bla*_IMP_ (14.28%), and *bla*_KPC_ (4.54%). The rate was the lowest for *bla*_VMP_ (1.29%). Regarding Qnr genes, 14.93% of the isolates carried *qnr*S, followed by *qnr*A (9.74%) and *qnr*B (7.79%), whereas *sul*1 and *sul*2 depicted resistance as 4.54% and 19.48%, respectively. Additionally, *tet*A and *tet*B detection rates were 26.62% and 18.18%, respectively. Lastly, for *mcr-*1, the distribution was 11.68%, and *mcr-*2 (0.64%).

*K. pneumoniae* (18) isolates showed the presence of different ARGs from various animal-derived foods. Starting with ESBLs, the identification patterns seen were as follows: *bla*_CTX-M_ (30.43%), *bla*_SHV_ (30.43%), *bla*_TEM_ (34.78%), *bla*_OXA_ (13.04%), and *bla*_CMY_ was not found. In MBLs, only *bla*_VMP_ showed positive results (4.34%). Likewise, in the case of Qnr genes, *qnr*S was found in 8.69% of the isolates, along with *qnr*A (13.04%) and *qnr*B (21.73%), whereas in *sul*1 (21.73%) and *sul*2 (30.43%), resistance was calculated. Furthermore, the resistance rate of *tet*A and *tet*B was 43.47% and 4.34%, respectively. Finally, the resistance pattern was13.04% in *mcr-1* and 4.34% in *acc*.

**Figure 4:**
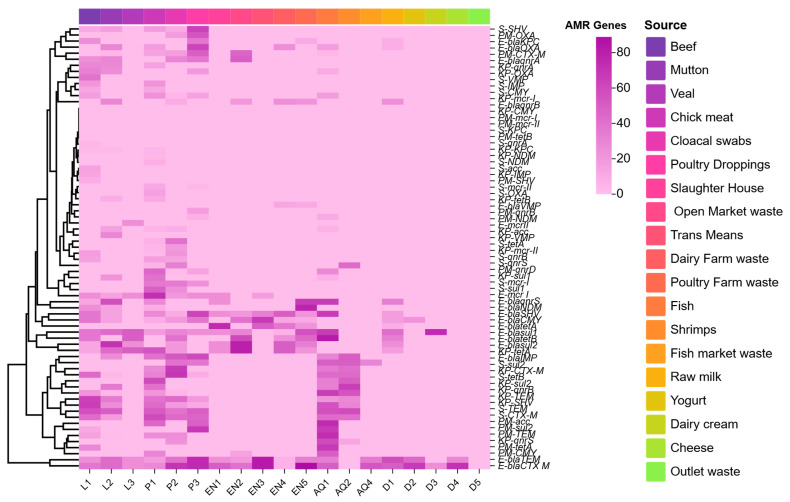
The cluster heat map depicts the distribution of various ARGs among Enterobacterales in the animal-derived foods. X-axis describing the sources of samples, which are mentioned in five animal-derived food categories like (livestock = L1, L2, L3; poultry = P1, P2, P3; environment EN1, EN2, EN3, EN4, EN5; aquatic productsAQ1, AQ2, AQ4; and dairy = D1, D2, D3, D4, D5), and sources depicting type/source of each category in colored block on the upper side. Regarding the Y-axis, the right side shows the type of ARGs like ESBLs (*bla*_CTX-M_, *bla*_TEM_, *bla*_SHV_, and *bla*_OXA_), MBLs (*bla*_NDM_, *bla*_KP_, *bla*_IMP_, and *bla*_VMP_), Quinolones (*qnr*_S_, *qnr*A, and *qnr*B), Tetracyclines (*tet*A and *tet*B), Sulphonamide (*sul*1 and *sul*2), Colistin (*mcr-1* and *mcr-2*), and aminoglycosides (*acc*) among Enterobacterales (*E. coli*, *K. pnumoniae*, *P. mirabilis*, and *Salmonella* spp.) The left side (tree) of the Y-axis illustrates the correlation of various ARGs and animal-derived food categories mentioned above. This similarity analysis was performed online at https://www.chiplot.online/ (accessed on 22 August 2025). This similarity indicated the correlation of the number and type of ARGs among Enterobacterales from animal-derived food sources.

**Figure 5:**
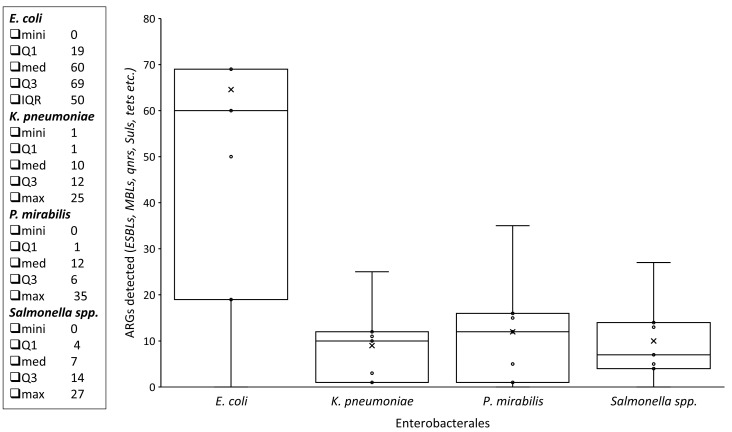
Box and Whisker plot displaying the distribution of various ARGs (y-axis), among isolated Enterobacterales (x-axis), along with various values (minimum, Q1, median, Q3, IQR, and maximum).

Presence of different ARGs in *P. mirabilis* (56) from various animal-derived foods. Regarding different ESBLs, the identification patterns seen were as follows: *bla*_CTXM_ (25%), *bla*_TEM_ (14.28%), *bla*_OXA_ (12.5%), and *bla*_CMY_ (10.71%), while *bla*_SHV_ was not found. In MBLs, only *bla*_NDM_ (1.78%) showed a positive result. In Qnr, *qnr*D was seen in 19.64% of isolates, along with *qnr*A and qnrB (3.57%), whereas, a 3.57% resistance rate was estimated in case of *sul*1. Additionally, the rate of *tet*A was 8.92% and it was absent in *tet*B. Lastly, the resistance rate was 21.42% in *acc*, while *mcr-1* and *mcr-2* were not detected (Figure 4 and Figure 5).

Lastly, among *Salmonella* spp. (35) isolates different ARGs were distributed as follows: *bla*_CTX-M_ (45.71%), *bla*_SHV_ (22.85%), *bla*_TEM_ (51.42%), *bla*_OXA_ (5.71%), and *bla*_CMY_ (2.85%). Regarding the detection rate of MBLs, *bla*_IMP_ and *bla*_VMP_ were (5.71%) and (2.85%), respectively. The lowest rate was for *bla*_OXA_ (2.85%) and *bla*_NDM_ was not detected, whereas *Qnr* genes, *qnr*S, were observed in 8.57% of the isolates, along with *qnr*B (5.71%). Additionally, the detection value of *sul*1, *sul*2, *tet*A, and *tet*B were 14.28%, 25.71%, 20% and 17.14%, respectively, whereas *mcr-1* was 17.14% and 2. 85% in *mcr-2*.

### 3.4. VAGs Among Enterobacterales Isolates

Overall, a significant distribution of various VAGs among Enterobacterales was estimated. Among the *E. coli* isolates pattern of various VAGs was observed, including *stx*1 (21.50%) and *stx*2 (30.76%), followed by enteropathogenic toxin *eae* (17.3%), hemolysin A *hyl*A (28.84%), and *iss* (25%). In case of toxins that cause virulence through fimbriae, there were *pap*A (1.92%) and *pap*C (7.69%), whereas *fim*H was found (26.92%) in the isolates. Regarding *tra*T (25%), while in *Omp*T and *Amp*C, resistance rate was detected as 21.5% and 3.83%, respectively.

The *K. pneumoniae* isolates showed the presence of different VAGs; among these, *mrp*A, *Wab*G, and *Uge* revealed detection rates of 83.33% and 75%, respectively, followed by *fim*H identification patterns, i.e., among 83.33% isolates. Likewise, VAGs involved in iron uptake were also detected including *Kfu*, with *ent*B and *iro*NB with 25% and 8.33% isolates, respectively, whereas *mrk*D was observed in 75% and *ure*A in 83.33% isolates. In the end, *all*S and *rmp*A were not detected in any of the isolates.

In case of *P. mirabilis* VAGs, a significant detection rate was recorded as *zap*A (39.53%), along with *uca*A (34.88%), *hpm*A (32.55%), respectively, whereas *ire*A was found in 30.23% isolates, followed by 27.9% in *mrp*A, 23.35% in *pt*A, and 20.93% isolates were detected in *atf*A. However, the lowest detection rate was observed in *fIi*L (9.3%) (Figure 4).

**Figure 6:**
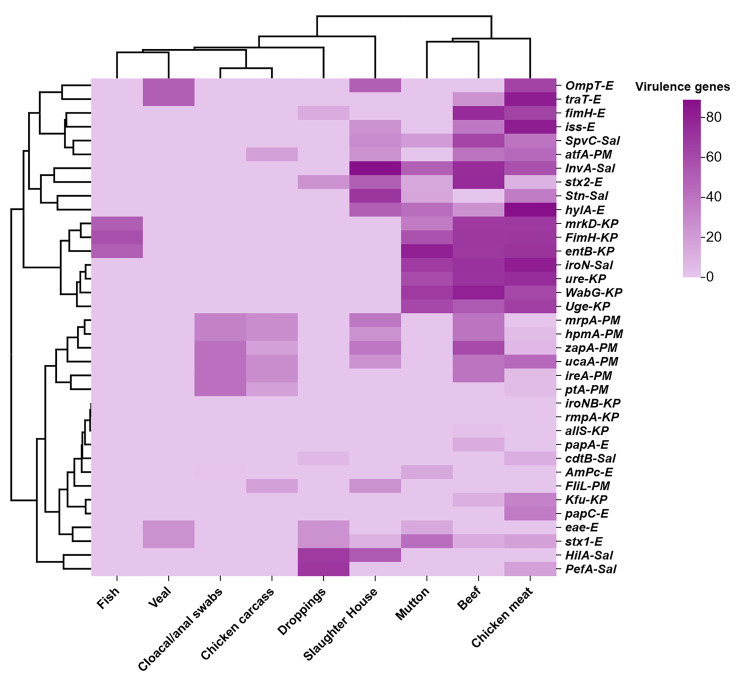
The cluster heat map describes the distribution of various VAGs among Enterobacterales in the animal-derived samples. X-axis depicts the animal-derived food categories like livestock (beef, mutton, and veal), poultry (chicken meat, chicken carcass, and cloacal swabs), environment (slaughterhouse), and aquatic products (fish). Regarding the Y-axis, the right side shows the type of VAGs like *stx*1, *stx*2, *eae*, hylA, *iss*, papA, pap*C*, *fim*H, *tra*T, *Omp*T, *Ampc* in *E. coli*, *fim*H, *mrk*D, *Wab*G, *uge*, *Kfu*, *ure*, *rmp*A, *ent*B, *iro*N, and *all*S in *K. pnumoniae*; *uca*A, *atf*A, *zap*A, *pt*A, *hpm*A, *ire*A, and *mrp*A in *P. mirabilis*; and *Fli*L, *Inv*A, *Spv*C, *pef*A, *cdt*B, *Stn*, *Hil*A, and *iro*N in *Salmonella* spp. This similarity analysis was performed online at https://www.chiplot.online/. This similarity indicated the correlation of the number and type of VAGs among Enterobacterales from animal-derived food sources.

The *Salmonella* spp. isolates were found to be positive for different VAGs. The *Inv*A was highest with a rate of 67.85%, followed by *iro*N (35.71%) and *Hil*A (32.14%). Likewise, detection rates of 28.57% in *Stn* and 25% in *Pef*A and *Spv*C, and 7.14% in *cdt*B, were also recorded (Figure 6).

### 3.5. Co-Existence of bla_NDM_ and mcr-1 Among Enterobacterales Isolated from Various Animal-Derived Foods

A total of 263 (29.06%) of Enterobacterales were observed for the co-occurrence of *bla*_NDM_ and *mcr-1* among various sources of animal-derived foods. Overall, 30 (11.40%) *bla*_NDM_ and 27 (10.26%) *mcr-1* exhibited interaction among various animal-derived food source categories. Firstly, in *E. coli*, the poultry products, particularly chicken meat, were at the top, showing higher interaction with 3:8 (*bla*_NDM_: *mcr*-*1*), whereas poultry dropping had 5:2, and cloacal swabs 1:2. Similarly, in livestock, the beef samples were depicting 3:2 and mutton had 1:1. Regarding environmental samples, various transport means described interaction 2:1 and slaughterhouses with 1:1. No significant interaction was seen in aquatic and dairy products. Meanwhile, among *K. pneumoniae*, *P. mirabilis*, and *Salmonella* spp. no such interaction was seen, but interestingly, various poultry products in *K. pnumoniae* and *Salmonella* spp., depicting a high *mcr-1* number (Table 2).

## 4. Discussion

Antimicrobial resistance (AMR) represents an escalating global health crisis that threatens human, animal, and environmental well-being, underscoring its intrinsic link to the One Health framework [14]. Primarily, the excessive use of antimicrobials in food-producing animals (FPAs) as feed additives is directly associated with AMR. This irrational use results in the emergence of resistant bacteria through selection pressure, which can be transmitted to humans directly or indirectly via contaminated food supply chain, water, soil, and environmental pathways, particularly when animal waste is used in agriculture and aquaculture [15], which makes it a significant One Health threat across the globe.

The specific objective of the proposed study was to estimate the distribution of MDR Enterobacterales among animal-derived food sources with emphasis on genetic diversity based on ARGs and VAGs. The findings exhibited a significant distribution of Enterobacterales, particularly *E. coli*, among study samples, which is a serious public health concern. Enterobacterales, particularly *E. coli*, serve as a primary public health threat due to their ubiquity and efficient horizontal gene transfer (HGT) capability, facilitating the rapid dissemination of MDR strains across ecological niches [16]. Moreover, VAGs, across different Enterobacterales, significantly influence the pathogenicity and endurance. [17]. Previously, a few studies from Pakistan also reported the distribution of MDR bacterial strains among animal-derived food, e.g., shiga toxin *E. coli* (STEC) non-O157 in raw milk [18]. Similar findings showing the distribution of STEC with unique resistance patterns against various antibiotics were reported from Khyber Pakhtunkhwa province of Pakistan [19].

The present findings revealed that an association exists between various VAGs and ARGs among Enterobacterales. It is a studied fact that microbes with diversified resistance patterns and ARGs showed a significant diversity among VAGs, and vice versa. A study published in the recent past documented a comprehensive analysis of bacterial genetic material and reported association patterns between some VAGs and AMR at diverse genetic locations in bacteria [20]. Moreover, in the present study, co-existence of *bla*_NDM_ and *mcr-1* among isolates; (<0.05) is also annoying, as carbapenems and colistin are considered as last resort antibiotics. Earlier, a study from China reported the steady existence of carbapenem and colistin resistance in *E. coli* among animal study sources. It was observed that diversity among isolates showed the co-existence of *bla*_NDM_ and *mcr-1.1* may be a risk cause of dissemination of resistant *E. coli* vertically or horizontally, i.e., HGT [21].

The misuse of antibiotics in FPAs and food processing significantly contributes to the dissemination of resistant strains, including Enterobacterales co-harboring *mcr-1* and *bla*_NDM_ [22]. As per the findings of the current study, the *E. coli* isolates exhibited significant colistin resistance. Likewise, distribution of colistin-resistant *E. coli* was reported in a recent study from Lahore, Pakistan, in which they found 78% colistin resistance among commercial poultry [23]. The distribution rate in this study was higher as compared to the findings of the current study. A possible reason for this distribution difference was the source of the samples, which were from commercial poultry only. This aligns with global trends as well: studies from different regions of the globe have reported the distribution rate of *mcr-1*-harboring *E. coli*, e.g., Malaysia 100%, Bangladesh 55.77%, and Peru 16% [24,25].

Selection pressure created by this irrational antimicrobial use remains a significant factor that causes the emergence of resistant pathogens like *K. pneumoniae*, which is an evolving foodborne resistant pathogen with an array of VAGs and ARGs. Consistent with previous findings from animal-derived foods [26], this study exhibited a range of ARGs and VAGs among isolates. This aligns with the pattern reported from China [27]. Conversely, livestock-associated data in Pakistan is limited; few reports showing a lower distribution rate have been documented [28]. The findings have been reported in a recent study from Pakistan showing a 15% distribution rate of *K. pnumoniae* among fish and poultry meat with a discrete resistance profile against various antibiotics. Additionally, the detection rate of the ARGs was also comparable with the findings of the present study [29]. Comparable resistance levels have been observed in neighboring countries, including India (56%) and Iran (59%) [30]. These results collectively emphasize how irrational antimicrobial use in FPAs facilitates the transmission of resistant bacterial strains along with ARGs, environmental contamination, and significant public health risks across the globe.

Though local data is limited, comparable findings have been reported showing distribution of resistant Enterobacterales, e.g., *P. mirabilis*, with a unique resistance pattern, was recovered from poultry carcasses, unlike the findings of the present study, in which a variety of sample sources were studied [31]. Similarly, results of the current study highlighted *Salmonella* as a significant One Health threat. The findings of the present study showed that fecal samples from livestock and poultry farms were the primary transmission source. Corroborating findings have been reported from various regions of Pakistan displaying *Salmonella* as a significant One Health threat associated with poultry and poultry products [32]. Moreover, similar resistance patterns and detection of various ARGs among *Salmonella* isolates have been reported in previous studies, particularly *mcr-1*-harboring *Salmonella* isolates among poultry specimens [33].

As per the results of the proposed study plan, a significant distribution of MDR Enterobacterales among animal-derived food sources emphasized the One Health significance. Moreover, resistance against some of the last resort antibiotics, such as carbapenems and colistin, is worrisome, as findings showed the detection of *bla*_NDM_ and *mcr-1*-harboring isolates recovered from different sample sources. MDR Foodborne Enterobacterales is associated with high morbidity and mortality worldwide. Reports from various regions of the world, especially of *bla*_NDM_ and *mcr-1*-harboring *E. coli* and *Salmonella*, affirmed the One Health significance of this menace [34,35]. A possible way to overcome this menace is to restrain the subtherapeutic and prophylactic use of such antibiotics, e.g., colistin. Several studies have reported different alternatives with potential antibacterial activity, which may be used as growth promoters instead of antibiotics. Additionally, such compounds, e.g., probiotics, phytogenic compounds, and antimicrobial peptides, have negligible depressing effects on animal microbiome, which would be beneficial to restrain selection pressure and resistant bacteria [36].

The findings of the proposed study represented the distribution of Enterobacterales among sampled populations rather than the overall food supply chain, as the sample collection was performed through convenient random sampling that is driven by market suitability and public demand. Additionally, seasonal and economic factors may also display an estimation of biased distribution. Likewise, due to limited funding, the current study has some limitations, like genetic characterization of the isolates by employing whole genome sequencing to find out the strains and serovars of the pathogens distributed among various specimens. Future studies harnessing cutting-edge molecular tools are needed to comprehend the molecular insights and genetic traits of the disseminated animal-derived food-borne pathogens with One Health significance.

## 5. Conclusions

Taking together, distribution of MDR Enterobacterales among animal-derived food, particularly *bla_NDM_* and *mcr-1*-harboring isolates, is a serious One Health concern. Poultry was recognized as the highest risk source, suggesting irrational use of antibiotics in commercial poultry fuels the dissemination of resistant pathogens, especially *E. coli.* Additionally, detection of resistant isolates among environmental sources demonstrated a significant risk as well; such sources need to be studied further to strengthen surveillance, as it is one of the neglected subjects, especially in farm animals, i.e., poultry and dairy. In the given scenario, strengthened surveillance and prudent antibiotic use in poultry and food-producing animals are key to slowing the resistance and dissemination of MDR pathogens.

## Figures and Tables

**Table 1:** Detailed category-wise distribution of Enterobacterales along with statistical analysis.

Enterobacterales	Sample Categories *																						
	Livestock				Poultry				Aquatic				Environment					Dairy					Total
	Bf	Mt	Vl	MS	mt	Sbs	CC	Dpg	Fis	Shri	Mkt	Trp	SH	OPW	TM	DFW	PFW	RM	YGT	Cr	CHS	OW	
*E. coli*	8	7	4	--	11	10	--	8	6	2	7	17	4	10	6	8	9	12	9	4	7	5	154
*K. pneumoniae*	2	3	--	--	5	2	--	3	2	1	--	--	--	--	--	--	--	--	--	--	--	--	18
*P. mirabilis*	5	4	--	8	7	12	11	--	4	--	5	--	--	--	--	--	--	--	--	--	--	--	56
*Salmonella* spp.	3	4	--	--	6	--	--	8	4	3	--	--	--	--	--	--	7	--	--	--	--	--	35
Total	18	18	4	8	29	24	11	19	16	6	12	17	4	10	6	8	16	12	9	4	7	5	263
% Distribution from positive samples (n = 263)	6.84	6.84	1.52	3.0	11.0	9.12	4.18	7.22	6.0	2.28	4.56	6.46	1.52	3.80	2.28	3.0	6.0	4.56	3.42	1.52	2.66	1.90	
Overall % Distribution (n = 905)	1.98	1.98	0.40	0.88	3.20	2.65	1.21	2.09	1.76	0.66	1.32	1.87	0.44	1.10	0.66	0.88	1.76	1.32	0.99	0.44	0.77	0.55	29%
**Statistical Analysis**																							
**Source of Variation**				**SS**					**df**			**MS**			**F**		***p*-value**				**F crit**		
Between Groups				881.16					21			41.96			1.71		0.037				1.637		
Within Groups				3242.96					132			24.57			1.64								
Total				4124.13					153			66.53			3.34								

* livestock: Bf = beef, Mt = mutton, Vl = veal, MS = manure sludge; poultry: mt = meat, Sbs = swabs, CC = chicken carcass, Dpg = droppings; aquatic: Fis = fish, Shri = shrimps, Mkt = market, Trp = transport; environment: SH = slaughterhouse, OPW = open market waste, TM = transport means, DFW = dairy farm waste, PFW = poultry farm waste; dairy: RW = raw milk, YGT = yogurt, cr = cream, CHS = cheese, OW = outlet waste.

**Table 2:** Co-existence of *bla*_NDM_ and *mcr-1* among Enterobacterales isolated from various animal-derived foods.

Isolates	Specimen Category	Samples Sources	Total Samples	Positive Samples	*bla*_NDM_	*mcr-1*	*p*-Value
*E. coli*	Livestock	Beef	25	8	3 (37.5%)	2 (25%)	<0.05
		Mutton	25	7	1 (14.28%)	1 (14.28%)	
		Veal	25	4	ND	1 (25%)	
	Poultry	Chicken meat	25	11	3 (27.27%)	8 (72.72%)	
		Cloacal/anal swabs	25	10	1 (10%)	2 (20%)	
		Droppings	25	8	5 (62.5%)	2 (25%)	
	Environment	Slaughterhouse	25	4	1 (25%)	1 (25%)	
		Open market waste	25	10	1 (20%)	ND	
		Transport Means	25	6	2 (33.33%)	1 (16.67%)	
		Dairy farm waste	25	8	4 (50%)	ND	
		Poultry farm waste	25	9	2 (22.22%)	ND	
	Aquatic	Fish	25	6	4 (66.67%)	ND	
		Shrimps	25	2	ND	ND	
		Market waste	25	7	ND	ND	
		Transport means	25	17	ND	ND	
	Dairy	Raw milk	25	12	2 (16.67%)	ND	
		Yogurt	25	9	ND	ND	
		Dairy cream	25	4	ND	ND	
		Cheese	25	7	ND	ND	
		Outlet waste	25	5	ND	ND	
*K. pneumoniae*	Livestock	Beef	15	2	ND	17.11	
		Mutton	15	3	ND	ND	
	Poultry	Chicken meat	20	5	ND	2 (23.21)	
		Cloacal swabs	15	2	ND	4.01	
		Poultry droppings	15	3	ND	1 (14.36)	
	Aquatic	Fish	10	2	ND	17.45	
		Shrimp	10	1	ND	ND	
*P. mirabilis*	Poultry	Chicken meat	25	7	ND	ND	
		Chicken carcass	25	11	ND	ND	
		cloacal/anal swabs	25	12	1 (8%)	ND	
	Livestock	Beef	25	5	ND	ND	
		Mutton	25	4	ND	ND	
		Manure sludge	25	8	ND	ND	
	Aquatic	Raw fish	25	4	0 (11.23%)	ND	
		Fish Market sludge/waste	25	5	ND	ND	
*Salmonella* spp.	Livestock	Beef	15	3	ND	ND	
		Mutton	15	4	ND	ND	
	Poultry	chicken meat	20	6	ND	2 (36.4)	
		poultry farm	15	7	ND	2 (34.67)	
		poultry droppings	20	8	ND	2 (23.45)	
	Aquatic	Fish	10	4	ND	ND	
		shrimp	10	3	ND	ND	
	**Total**		**905**	**263**	**30**	**27**	

## Data Availability

The original contributions presented in this study are included in the article/Supplementary Material. Further inquiries can be directed to the corresponding authors.

## Supplementary Materials

The following supporting information can be downloaded at: https://www.mdpi.com/article/10.3390/pathogens14101040/s1, Tables S1–S4: Details of primers used in the study.

## Abbreviations

The following abbreviations are used in this manuscript:

MALDI-TOF	Matrix-assisted laser desorption/ionization-time of flight
ARGs	Antibiotic resistance genes
VAGs	Virulence-associated genes

## References

[1] Hernando-Amado S., Coque T.M., Baquero F., Martínez J.L. (2019). Defining and combating antibiotic resistance from One Health and Global Health perspectives. Nat. Microbiol..

[2] Gros M., Mas-Pla J., Sànchez-Melsió A., Čelić M., Castaño M., Rodríguez-Mozaz S., Borrego C.M., Balcázar J.L., Petrović M. (2023). Antibiotics, antibiotic resistance and associated risk in natural springs from an agroecosystem enviorment. Sci. Total. Environ..

[3] Jonas T., Trethewey B. (2023). Agroecology for Structural One Health. Development.

[4] Morton S., Pencheon D., Squires N. (2017). Sustainable Development Goals (SDGs), and their implementation: A national global framework for health, development and equity needs a systems approach at every level. Br. Med. Bull..

[5] Gilbert P., Brown M.R. (2018). Screening for novel antimicrobial activity/compounds in the pharmaceutical industry. Microbiological Quality Assurance.

[6] Mulchandani R., Tiseo K., Nandi A., Klein E., Gandra S., Laxminarayan R., Van Boeckel T. (2025). Global trends in inappropriate use of antibiotics, 2000–2021: Scoping review and prevalence estimates. BMJ Public Health.

[7] Schar D., Klein E.Y., Laxminarayan R., Gilbert M., Van Boeckel T.P. (2020). Global trends in antimicrobial use in aquaculture. Sci. Rep..

[8] Okocha R.C., Olatoye I.O., Adedeji O.B. (2018). Food safety impacts of antimicrobial use and their residues in aquaculture. Public Health Rev..

[9] Mohsin M., Van Boeckel T.P., Saleemi M.K., Umair M., Naseem M.N., He C., Khan A., Laxminarayan R. (2019). Excessive use of medically important antimicrobials in food animals in Pakistan: A five-year surveillance survey. Glob. Health Action.

[10] McEwen S.A., Collignon P.J. (2018). Antimicrobial resistance: A one health perspective. Microbiol. Spectr..

[11] Sarwar A., Aslam B., Rasool M.H., Bekhit M.M.S., Sasanya J. (2024). A Health Threat from Farm to Fork: Shiga Toxin-Producing *Escherichia coli* Co-Harboring *bla*_NDM-1_ and *mcr-1* in Various Sources of the Food Supply Chain. Pathogens.

[12] Ferreira M., Leão C., Clemente L., Albuquerque T., Amaro A. (2022). Antibiotic Susceptibility Profilesand Resistance Mechnasims of β-Lactams and Polymyxins of Escherchia coli from Briolers Raised under Intensive and Extensive Production Systems. Microorganisms.

[13] Huang H.-Y., Wang C.-F., Lu P.-L., Tseng S.-P. (2021). Clinical impact of the revised 2019 CLSI levofloxacin breakpoints in patients with Enterobacterales bacteremia. Antimicrob. Agents Chemother..

[14] Li X., Mowlaboccus S., Jackson B., Cai C., Coombs G.W. (2024). Antimicrobial resistance among clinically significant bacteria in wildlife: An overlooked one health concern. Int. J. Antimicrob. Agents.

[15] Singer A., Stanton I.C., Tipper H.J., Read D.S. (2021). Environment and Rural Affairs Monitoring & Modelling Programme-ERAMMP Report-55: Evidence Review on the Entry and Spread of Antimicrobial Resistance (AMR) in the Rural Water Environment in Wales.

[16] Tang F., Li C., Li R., Xi L., Wang F., Tian J., Luo W. (2024). Antibiotic-resistance profiles and genetic diversity of Shigella isolates in China: Implications for control strategies. Foodborne Pathog. Dis..

[17] Nwike I.E., Ugwu M.C., Ejikeugwu P.C., Ujam N.T., Iroha I.R., Esimone C.O. (2023). Phenotypic and molecular characterization of enteropathogenic *Escherichia coli* and *Salmonella* spp. causing childhood diarrhoea in Awka, South-Eastern Nigeria. Bull. Natl. Res. Cent..

[18] Ullah S., Khan S.U.H., Khan M.J., Khattak B., Fozia F., Ahmad I., Wadaan M.A., Khan M.F., Baabbad A., Goyal S.M. (2024). Multiple-Drug Resistant Shiga Toxin-Producing *E. coli* in Raw Milk of Dairy Bovine. Trop. Med. Infect. Dis..

[19] Ullah S., Khan S.U.H., Ali T., Zeb M.T., Riaz M.H., Khan S., Goyal S.M. (2024). Molecular characterization and antibiotic susceptibility of Shiga toxin-producing Escherichia coli (STEC) isolated from raw milk of dairy bovines in Khyber Pakhtunkhwa, Pakistan. PLoS ONE.

[20] Darmancier H., Domingues C.P.F., Rebelo J.S., Amaro A., Dionísio F., Pothier J., Serra O., Nogueira T. (2022). Are Virulence and Antibiotic Resistance Genes Linked? A Comprehensive Analysis of Bacterial Chromosomes and Plasmids. Antibiotics.

[21] Guan Y., Wang Z., Shang Z., Zou H., Zhao L., Hou X., Wu T., Meng M., Li X. (2024). Steady existence of *Escherichia coli* co-resistant to carbapenem and colistin in an animal breeding area even after the colistin forbidden. J. Environ. Manag..

[22] Ibrahim S., Hoong L.W., Siong Y.L., Mustapha Z., Zalati C.W.S.C.W., Aklilu E., Mohamad M., Kamaruzzaman N.F. (2021). Prevalence of antimicrobial resistance (AMR) *Salmonella* spp. and *Escherichia coli* isolated from broilers in the East Coast of Peninsular Malaysia. Antibiotics.

[23] Bastidas-Caldes C., Cisneros-Vásquez E., Zambrano A., Mosquera-Maza A., Calero-Cáceres W., Rey J., Yamamoto Y., Yamamoto M., Calvopiña M., de Waard J.H. (2023). Co-harboring of beta-lactamases and mcr-1 genes in *Escherichia coli* and *Klebsiella pneumoniae* from healthy carriers and backyard animals in rural communities in Ecuador. Antibiotics.

[24] Huda M.N.U., Shabbir M.Z., Tahir A.H., Ahmad A.A., Bin Zahoor U., Ahmad W., Raza A., Afzal F., Khalid A.R., Shabbir M.A.B. (2025). Molecular detection and prevalence of colistin-resistant *Escherichia coli* in poultry and humans: A one health perspective. Braz. J. Microbiol..

[25] Zhang S., Yang G., Ye Q., Wu Q., Zhang J., Huang Y. (2018). Phenotypic and genotypic characterization of *Klebsiella pneumoniae* isolated from retail foods in China. Front. Microbiol..

[26] Wu X., Liu J., Feng J., Shabbir M.A.B., Feng Y., Guo R., Zhou M., Hou S., Wang G., Hao H. (2022). Epidemiology, environmental risks, virulence, and resistance determinants of *Klebsiella pneumoniae* from dairy cows in Hubei, China. Front. Microbiol..

[27] Kar M., Siddiqui T., Sengar S., Sahu C. (2024). A comparative in vitro sensitivity study of “cefepime-tazobactam” and otherantibiotics against Gram-negative isolates from intensive care unit. J. Lab. Physician.

[28] Theocharidi N.A., Balta I., Houhoula D., Tsantes A.G., Lalliotis G.P., Polydera A.C., Stamatis H., Halvatsiotis P. (2022). High prevalence of *Klebsiella pneumoniae* in Greek meat products: Detection of virulence and antimicrobial resistance genes by molecular techniques. Foods.

[29] Qamar M.U., Fizza K., Chughtai M.I., Shafique M., Seytkhanova B., Yktiyarov A., Aatika, Saleem Z., Mustafa S., Tufail Z. (2025). Food Safety Concerns in Pakistan: Monitoring of Antimicrobial-Resistant Bacteria and Residue Contamination in Commercially Available Fish and Poultry Meat Samples. Foodborne Pathog. Dis..

[30] Mohammed A.B., Anwar K.A. (2022). Phenotypic and genotypic detection of extended spectrum beta lactamase enzyme in *Klebsiella pneumoniae*. PLoS ONE.

[31] Ishaq K., Ahmad A., Rafique A., Aslam R., Ali S., Shahid M.A., Sarwar N., Aslam M.A., Aslam B., Arshad M.I. (2022). Occurrence and antimicrobial susceptibility of Proteus mirabilis from chicken carcass. Pak. Vet. J..

[32] Tagar S., Qambrani N.A. (2023). Bacteriological quality assessment of poultry chicken meat and meat contact surfaces for the presence of targeted bacteria and determination of antibiotic resistance of *Salmonella* spp. in Pakistan. Food Control.

[33] Mahmood S., Rasoo M.H., Khurshid M., Aslam B. (2025). Genetic outlook of Colistin resistant *Salmonella enterica* Serovar Typhimurium recovered from Poultry-Environment Interface: A One Health Standpoint. Pak. Vet. J..

[34] Fu B., Xu J., Yin D., Sun C., Liu D., Zhai W., Bai R., Cao Y., Zhang Q., Ma S. (2024). Transmission of bla NDM in Enterobacteriaceae among animals, food and human. Emerg. Microbes Infect..

[35] Mladenović K.G., Grujović M.Ž., Kiš M., Furmeg S., Tkalec V.J., Stefanović O.D., Kocić-Tanackov S.D. (2021). Enterobacteriaceae in food safety with an emphasis on raw milk and meat. Appl. Microbiol. Biotechnol..

[36] Wang F., Zhang W., Niu D. (2021). foodborne Enterobacteriaceae of animal origin. Front. Cell. Infect. Microbiol..

