# Supplementary material for: Genetic Diversity and Antibiotic Resistance Paradigm of Enterobacterales in Animal-Derived Food Sources: A One Health Disquiet
Source: Pathogens. 2025 Oct 13;14(10):1040. doi: 10.3390/pathogens14101040 (PMC12566681; doi:10.3390/pathogens14101040)
Supplement: Supplementary file 1 [file pathogens-14-01040-s001.zip › pathogens-3903725-supplementary.pdf]

Table S1: Detail of Primers used in the study *Enterobacterales* (*E. coli*)

Antibiotics/VAGs	Target	Sequence	Annealing Temp (°C)	Amplicon Size
		**ARGs**		
β-lactams	*bla*CTX-M-1	F: ATGTGCAGYACCAGTAARGTKATGGC<br>R: TGGGTRAARTARGTSACCAGAAYCAGCGG	61	593
	*bla*TEM	F: CGCCGCATACACTATTCTCAGAATGA<br>R: ACGCTCACCGGCTCCAGATTTAT	61	445
	*bla*SHV	F: CTTTATCGGCCCTCACTCAA<br>R: AGGTGCTCATCATGGGAAAG	61	237
	*bla*CMY	F: CTGAC AGCCT CTTTC TCCAC A<br>R: CTACG TAGCT GCCAA ATCCA C	63	1100
Carbapenems	*bla*NDM-1	F: AACGCATTGGCATAAGTCGC<br>R: AACGCATTGGCATAAGTCGC	59	292
	*bla*0XA	F: TTGGTGGCATCGATTATCGG<br>R: GAGCACTTCTTTTGTGATGGC	55	743
	blaKPC	F: TGCAGAGCCCAGTGTCAGTTT<br>R: CGCTCTATCGGCGATACCA	52	880
	blaIMP	F: GGAATAGAGTGGCTTAATTCTC<br>R: CCAAACCACTACGTTATC	54	624
	blaVIM	F: GTTTGGTCGCATATCGCAAC<br>R: AATGCGCAGCACCAGGATAG	52	389
Quinolones	*qnr*A	F: TCAGCAAGAGGATTTCTCA<br>R: GGCAGCACTATTACTCCCA	51	627
	*qnr*B	F: CGACCTGAGCGGCACTGAAT<br>R: TGAGCAACGATGCCTGGTAG	52	515
	*qnr*S	F: GCCAATTGYTACGGKATWGAG<br>R: GACTCTTTCARTGATGCRCC	54	227
Sulphonamides	*sul*1	F: TCA CCG AGG ACT CCT TCT TC<br>R: CAG TCC GCC TCA GCA ATA TC	53	435
	*sul*2	F: GCGCTCAAGGCAGATGGCAT<br>R: GCGTTTGATACCGGCACCCT	53	293
Tetracycline	*tet*A	F: GTAATTCTGAGCACTGTCGC<br>F: GTAATTCTGAGCACTGTCGC	55	956
	*tet*B	F: CTCAGTATTCCAAGCCTTTG<br>R: ACTCCCCTGAGCTTGAGGGG	55	414
Colistin	*mcr*-I	F: CGGTCAGTCCGTTTGTTC<br>R: CTTGGTCGGTCTGTAGGG	51	302
	mcr-II	F: ATGACCTTGCGATGCTCTATGA<br>R: CGAATGCCTGGCGTGTTT	56	309
Aminoglycosides	*aad*A1	F: TAT CCA GCT AAG CGC GAA CT<br>R: ATT TGC CGA CTA CCT TGG TC	58	447
	aac-3-IV	F: CTT CAG GAT GGC AAG TTG GT<br>R: TCA TCT CGT TCT CCG CTC AT	58	286
	*aph*A-1	F: AACGTCTTGCTCGAGGCCGCG<br>R: GGCAAGATCCTGGTATCGGTCTGC	65	670
		**VAGs**		
Shiga toxin	*stx1*	F: CACAATCAGGCGTCGCCAGCGCACTTGCT<br>R: TGTTGCAGGGATCAGTGGTACGGGGATGC	58	660
	stx2	F: CCACATCGGTGTCTGTTATTAACCACACC<br>R: GCAGAACTGCTCTGGATGCATCTCTGGTC	58	372
Enteropathogenic toxin	*eae*	F: CCCGAATTCGGCACAAGCATAAGC<br>R: CCCGGATCCGTCTCGCCAGTATTCG	57	881
Hemolysin F	*hylF*	F: GGCCACAGTCGTTTAGGGTGCTTACC<br>R: GGCGGTTTAGGCATTCCGATACTCAG	58	450
Increased serum survival	*Iss*	F: CAGCAACCCGAACCACTTGATG<br>R: AGCATTGCCAGAGCGGCAGAA	57	323
Type 1 fimbrin D mannose adhesion	*fimH*	F: TCGAGAACGGATAAGCCGTGG<br>R: GCAGTCACCTGCCCTCCGGTA	58	508
P fimbriae	*papC*	F: TGATATCACGCAGTCAGTAGC	57	501

		R: CCGGCCATATTCACATAA		
F factor transfer gene	*traT*	F: GGTGTGGTGCGATGAGCACAG<br>R: CACGGTTCAGCCATCCCTGAG	64	290
outer membrane protein	*Ompt*	F: TCATCCCGGAAGCCTCCCTCACTACTAT<br>R: TAGCGTTTGCTGCACTGGCTTCTGATAC	59	496
ampicillin resistance gene group C	*Ampc*	F: GTGAAGCCRTCTGGTTTGAG<br>R: GCGACATAGCTACCAAATCCG	63	494

Table S2: Detail of Primers used in the study (*K. pneumoniae*)

Antibiotics/VAGs	Target	Sequence	Annealing Temp (°C)	Amplicon Size
		**ARGs**		
β-lactams	*bla*CTX-M-1	F: TTAGGAAATGTGCCGCTGTA<br>R: CGATATCGTTGGTGGTACCAT	56	688
	*bla*TEM	F: CATTTCCGTGTCGCCCTTATTC<br>R: CGTTCATCCATAGTTGCCTGAC	50	800
	*bla*SHV	F: AGCCGCTTGAGCAAATTAAAC<br>R: ATCCCGCAGATAAATCACCAC	58	713
	*bla*OXA	F: GGCACCAGATTCAACTTTCAAG<br>R: GACCCCAAGTTTCCTGTAAGTG	60	564
Carbapenems	*bla*NDM-1	F: GGTTTGGCGATCTGGTTTTC<br>R: CGGAATGGCTCATCACGATC	52	621
	*bla*OXA	F: TGA GCA AGT TAT CTG TAT TC<br>R: TTA GTT GCT TGG TTT TGA TG	55	139
	blaKPC	F: GAT GGT GTT TGG TCG CAT A<br>R: CGA ATG CGC AGC ACC AG	55	390
	blaIMP	F: ATG TCA CTG TAT CGC CGT CT<br>R: TTT TCA GAG CCT TAC TGC CC	55	538
	blaVIM	F: TTG GTG GCA TCG ATT ATC G<br>R: GAG CAC TTC TTT TGT GAT GGC	57	281
Quinolones	*qnr*A	F: ATTTCTCACGCCAGGATTTG<br>R: GATCGGCAAAGGTTAGGTCA	55	627
	*qnr*B	F: GATCGTGAAAGCCAGAAAGG<br>R: ACGATGCCTGGTAGTTGTCC	55	469
	*qnr*D	F: CGAGATCAATTTACGGGGAATA<br>R: AACAAGCTGAAGCGCCTG	55	523
Sulphonamides	*sul*1	F： GTGACGGTGTTCGGCATTCT<br>R：TCCGAGAAGGTGATTGCGCT	55	779
	*sul*2	F： CGGCATCGTCAACATAACCT<br>R：TGTGCGGATGAAGTCAGCTC	55	721
Tetracycline	*tet*A	F: GTAATTCTGAGCACTGTCGC<br>R: CTGCCTGGACAACATTGCTT	56	956
	*tet*B	F: CTCAGTATTCCAAGCCTTTG<br>R: ACTCCCCTGAGCTTGAGGGG	56	415
Colistin	*mcr*-I	F: ATCCCATCGCGGACAATCTC<br>R: AGACCGTGCCATAAGTGTCA	60	177
	mcr-II	F: GTGTCAGCCTTGTGYTGTTG<br>R: ATCGGCGTAATCGGRTTRAT	60	112
Aminoglycosides	*aac* (6')-ib-cr	F: TTGCGATGCTCTATGAGTGGCTA<br>R: CTCGAATGCCTGGCGTGTTT	57	482
	aac	F: ATGACCTTGCGATGCTCTATGA<br>R: CGAATGCCTGGCGTGTTT	58	286
		**VAGs**		
Type 1 fimbrin D mannose adhesion	*fimH*	F: GCTCTGGCCGATACCACCACGG<br>R: GCGAAGTAACGTGCCTGGAACGG	58	423
	allS	F: CATTACGCACCTTTGTCAGC<br>R: GAATGTGTCGGCGATCAGCTT	58	764
	ureA	F: GCTGACTTAAGAGAACGTTATG<br>R: GATCATGGCGCTACCTCTTAAA	57	337
	*wab*G	F: CTCTGGTGCGGCAGAAGTAC<br>R: TGGCCGTCGACGATAAACTC	58	340
	*mrk*D	F: AAGCTATCGCTGTACTTCCGGCA<br>R: GGCGTTGGCGCTCAGATAGG	57	960

	*iro*NB	F: GGCTACTGATACTTGACTATTC<br>R: CAGGATACAATAGCCCATAG	58	992
	Kfu	F: GGCCTTTGTCCAGAGCTACG<br>R: GGGTCTGGCGCAGAGTATGC	57	638
	*rmp*A	F: ACCCTTTACAGCCAAATTTTCTTGT<br>R: CTGGGCTACCTCTGCTTCATAT	64	468
	*ent*B	F: ATTTCCTCAACTTCTGGGGC<br>R: AGCATCGGTGGCGGTGGTCA	59	371
	uge	F: TCTTCACGCCTTCCTTCACT<br>R GATCATCCGGTCTCCCTGT	63	534

Table S3: **Detail of Primers used in the study (*P. mirabilis*)**

Antibiotics/VAGs	Target	Sequence	Annealing Temp (°C)	Amplicon Size
		**ARGs**		
β-lactams	*bla*CTX-M-1	F: TCAAGCCTGCCGATCTGGT<br>R: TGATTCTCGCCGCTGAAG	55	561
	*bla*TEM	F: GGGGATGAGTATTCAACATTTCC<br>R: GGGCAGTTACCAATGCTTAATCA	55	861
	*bla*0XA	F: TTGAAGGAACTGAAGGTTGT<br>R: CCAAGTTTCCTGTAAGTGCG	55	651
	*bla*CMY	F: TGGCCGTTGCCGTTATCTAC<br>R: CGTTAACGGCACGATGAC	63	868
Carbapenems	*bla*NDM-1	F: AACGCATTGGCATAAGTCGC<br>R: AACGCATTGGCATAAGTCGC	58	178
Quinolones	*qnr*D	F: CGAGATCAATTTACGGGGAATA<br>R: AACAAGCTGAAGCGCCTG	65	572
	*qnr*B	F: GATCGTGAAAGCCAGAAAGG<br>R: ATGAGCAACGATGCCTGGTA	65	476
	*qnr*A	F: CCAGGATTTGAGTGACAGC<br>R: TCCCAAGGGTTCCAGCA	65	592
Sulphonamides	*sul*1	F: GTGACGGTGTTCCGCA TTCT	72	779
	*sul*2	F: CATCATTTTCGGCATCGTC<br>R: TCTTGCGGTTTCTTTCAGC	72	793
Tetracycline	*tet*A	F: : GCTACATCCTGCTTGCCTTC<br>R: : CATAGATCGCCGTGAAGAGG	63	210
	*tet*B	F: TTGGTTAGGGGCAAGTTTTG<br>R: GTAATGGGCCAATAACACCG	63	406
Colistin	*mcr*-I	F: CGGTCAGTCCGTTTGTTC<br>R: CTTGGTCGGTCTGTAGGG	51	309
Aminoglycoside	*Acc*	F: ATGACCTTGCGATGCTCTATGA<br>R: CGAATGCCTGGCGTGTTT	54	486
		VAGs		
Fimbrae	*uca*A	F: GCTTTTACATCCCCAGCGGT<br>R: GCTGCATTTGCTGGCTCATC	60	476
	*atf*A	F: CATAATTTCTAGACCTGCCCTAGCA<br>R: CTGCTTGGATCCGTAATTTTTAACG	50	382
	*mrp*A	F: ATTTCAGGAAACAAAAGATG<br>R: TTCTTACTGATAAGACATTG	57	881
	*hpm*A	F: GTTGAGGGGCGTTATCAAGAGTC<br>R: GATAACTGTTTTGCCCTTTTGTGC	55	709
Protease	*zap*A	F: TGGCGCAAATACGACTACCA<br>R: TATCGTCTCCTTCGCCTCCA	57	323
	*pt*A	F: CCACTGCGATTATCCGCTCT<br>R: ATCGGCAGAAGTGACAAGCA	60	686
Siderophores	*ire*A	F: AAAGGGCGAGCGATTATGTATGG<br>R: ATTGGCGCTATGTTTTGGTGTCA	55	387
Flagella	*FliL*	F: CTCTGCTCGTGGTGGTGTCG<br>R: GCGTCGTCACCTGATGTGTC	57	770

Table S4: Detail of Primers used in the study (*Salmonella spp.*)

Antibiotics/VAGs	Target	Sequence	Annealing Temp (°C)	Amplicon Size
		**ARGs**		
β-lactams	*bla*CTX-M-1	F: GAGTTTCCCCATTCCGTTTC<br>R: CAGAATAAGGAATCCCATGGTT	54	909
	*bla*TEM	F: ATGAGTATTCAACATTTCCG<br>R: ACCAATGCTTAATCAGTGAG	54	964
	*bla*SHV	F: CACTCAAGGATGTATTGTG<br>R: TTAGCGTTGCCAGTGCTCG	55	807
	*bla*OXA	F: ACCAGATTCAACTTTCAA<br>R: TCTTGGCTTTTATGCTTG	55	590
Carbapenems	*bla*NDM-1	F: GGT TTG GCG ATC TGG TTTTC<br>R : CGG AAT GGC TCA TCA CGA TC	55	621
	*Bla*IMP	F: CTACCGCAGCAGAGTCTTTG<br>R: AACCAGTTTTGCCTTACCAT	54	587
	*bla*VIM	F: AGTGGTGAGTATCCGACAG<br>R: ATGAAAGTGCGTGGAGAC	58	261
	*bla*KPC	F: CTTGCTGCCGCTGTGCTG<br>R: GCAGGTTCCGGTTTTGTCTC	56	489
	*bla*OXA	F: GGACATAATCAGGTGATTCA<br>R: TAGATGCCGGCATTTCTGAC	53	670
Quinolones	*qnr*A	F: CCGCTTTTATCAGTGTGACT<br>R: ACTCTATGCCAAAGCAGTTG	55	188
	*qnr*B	F: GATCGTGAAAGCCAGAAAGG<br>R: ACGATGCCTGGTAGTTGTCC	54	469
	*qnr*D	F: CGAGATCAATTTACGGGGAATA<br>R: AACAAGCTGAAGCGCCTG	55	582
Sulphonamides	*sul*1	F: CGG CGT GGG CTA CCT GAA CG<br>R: GCC GAT CGC GTG AAG TTC	66	433
	*sul*2	F: CGG CAT CGT CAA CAT AAC CT<br>R: TGT GCG GAT GAA GTC AGC TC	66	721
Tetracycline	*tet*A	F-GTAATTCTGAGCACTGT<br>R- CCTGGACAACATTGCTT	45	954
	*tet*B	F- ACGTTACTCGATGCCAT<br>R-AGCACTTGTCTCCTGTT	48	1170
Colistin	*mcr*-I	F: CAGTATGGGATTGCGCAATGATT<br>R: TTATCCATCACGCCTTTTGAGTC	51	1197
	mcr-II	F: TATCGCTATGTGCTAAAGCCTG<br>R: AAAATACTGCGTGGCAGGTAGC	56	816
Aminoglycosides	*aad*A2	F: CGGTGACCATCGAAATTTCG<br>R: CTATAGCGCGGAGCGTCTCGC	54	250
	aac-3-IV	F: TGCTGGTCCACAGCTCCTTC<br>R: CGGATGCAGGAAGATCAA	63	653
	*aad*B	F: GAGGAGTTGGACTATGGATT<br>R: CTTCATCGGCATAGTAAAAG	55	208
		**VAGs**		
	*Inv*A	F: ACAGTGCTCGTTACGACCTGAA<br>R: AGACGACTGGTACTGATCGATAA	58	244
	*Spv*C	F: ACTCCTTGCACCAACCAAATGCGGA<br>R: TGTCTTCTTGCATTTCGCCACCATCA	58	571
	*Pef*A	F: TGTTTCCGGGCTTGTGCT<br>R: CAGGGCATTTGCTGATTCTTCC	57	700
	*cdt*B	F: GAAGCCGTTTATTTTGTAGAGGAGATGTT<br>R: ACAACTGTCGCATCTCGCCCCGTCATT	58	265
	Stn	F: TTGTGTCTATCACTGGCAACC<br>R: ATTCGTAACCCGCTCTCGTCC	57	617
	*Hil*A	F: CTGCCGCAGTGTTTAAGGATA<br>R: CTGTCGCCTTAATCGCAT	58	497
	iroN	F: ACTGGCACGGCTCGCTGTCGCTCTAT<br>R: CGCTTTACCGCCGTTCTGCCACTGC	57	1205
